# Reduction in Radiation Exposure in Minimally Invasive Pedicle Screw Placement Using a Tubular Retractor: A Pilot Study

**DOI:** 10.3390/tomography9010002

**Published:** 2022-12-20

**Authors:** Takeshi Oki, Alan Kawarai Lefor, Kentaro Nakamura, Takahiro Higashi, Isao Oki

**Affiliations:** 1 Department of Orthopedic Surgery, Yuki Hospital, 9629-1 Yuki, Yuki-City 307-0001, Ibaraki, Japan; 2 Department of Surgery, Division of Gastroenterological, General and Transplant Surgery, Jichi Medical University, 3311-1 Yakushiji, Shimotsuke-City 329-0498, Tochigi, Japan; 3 Department of Orthopedic Surgery, Shin Oyama City Hospital, 2251-1 Hitotonoya, Oyama-City 323-0827, Tochigi, Japan

**Keywords:** radiation protection, minimally invasive spine stabilization (MISt), percutaneous pedicle screws (PPS)

## Abstract

Background and Objectives: Percutaneous pedicle screw (PPS) placement is a minimally invasive spinal procedure that has been rapidly adopted over the last decade. However, PPS placement has elicited fear of increased radiation exposure from some surgeons, medical staff, and patients. This is because PPS placement is performed using a K-wire, and the operator must perform K-wire insertion into the pedicle under fluoroscopy. In order to prevent erroneous insertion, there are many occasions when direct insertion is required during radiation exposure, and the amount of radiation exposure to hands and fingers in particular increases. Although these problems are being addressed by navigation systems, these systems are still expensive and not widely available. Attempts have been made to address this situation using instrumentation commonly used in spinal surgery. First, it was considered to visualize anatomical bone markers using a tubular retractor and a microscope. In addition, the use of a self-drilling pin was adopted to locate the pedicle in a narrower field of view. Based on these considerations, a minimally invasive and highly accurate pedicle screw placement technique was developed while avoiding direct radiation exposure. This study evaluated radiation exposure and accuracy of pedicle screw placement using this new procedure in one-level, minimally invasive, transforaminal lumbar interbody fusion (MIS-TLIF). Materials and Methods: Data were collected retrospectively to review pedicle screw placement in single-level MIS TLIFs using a tubular retractor under a microscope. The total fluoroscopy time, radiation dose, and screw placement accuracy were reviewed. Extension of operating time was also evaluated. Results: Twenty-four patients underwent single-level MIS TLIFs, with placement of 96 pedicle screws. There were 15 females and 9 males, with an average age of 64.8 years and a mean body mass index of 25.5 kg/m^2^. The mean operating time was 201.8 min. The mean fluoroscopic time was 26.8 s. The mean radiation dose of the area dose product was 0.0706 mGy∗m^2^. The mean radiation dose of air kerma was 6.0 mGy. The mean radiation dose of the entrance skin dose was 11.31 mGy. Postoperative computed tomography scans demonstrated 93 pedicle screws confined to the pedicle (97%) and three pedicle screw breaches (3.2%; two lateral, one medial). A patient with screw deviation of the medial pedicle wall developed right-foot numbness necessitating reoperation. There were no complications after reoperation. The average added time with this combined procedure was 39 min (range 16–69 min) per patient. Conclusions: This novel pedicle screw insertion technique compares favorably with other reports in terms of radiation exposure reduction and accuracy and is also useful from the viewpoint of avoiding direct radiation exposure to hands and fingers. It is economical because it uses existing spinal surgical instrumentation.

## 1. Introduction

In recent years, occupational radiation exposure has been attracting attention due to related health risks during orthopedic surgery [1,2,3]. The implementation of percutaneous pedicle screw (PPS) placement has generated concern regarding the increased radiation exposure faced by surgeons, medical staff, and patients, especially during spinal surgery [4,5,6]. Advances in computed tomography (CT) scan-guided navigation and robotic surgery are expected to provide solutions to this problem [7,8]. While there are reports of reductions in radiation exposure, techniques to achieve this are not widely used due to their increased costs and extended operating time [9,10,11]. Therefore, it is expected that PPS placement using fluoroscopy will continue in the future.

Even in these situations, the surgeon’s interest is focused on the accuracy of the PPS placement, and little attention has been paid to fluoroscopic radiation exposure due to greater concern regarding neurovascular injury, visceral injury, and vertebral body fractures due to deviations of the Jamshidi needles, K-wires, and screws in the operating room [12,13,14,15]. However, both improving the accuracy of PPS placement and reducing radiation exposure must be addressed. There are some reports of reducing radiation exposure during PPS placement under fluoroscopy [16,17,18]. Clark et al. reported a reduction in radiation exposure (a mean fluoroscopic time of 10.43 s and a mean radiation dose of 0.295 mGy∗m^2^) by changing fluoroscopy settings and adjusting image resolution [19,20]. However, direct radiation exposure to hands and fingers during surgery remains a major problem. Yamashita et al. report in detail the amount of fluoroscopic radiation exposure during spinal examinations. It is noteworthy that the level of exposure encountered by the fingers near the exposure area is high (the total occupational radiation exposure doses received at the finger for a 3-month study period was 368 mSv) [21,22]. Bindal et al. reported an average exposure time of 1.69 min per operative procedure, an average skin surface exposure of 59.5 mSv for patients irradiated with posteroanterior fluoroscopy, and an average exposure of 0.76 mSv for the surgeon’s dominant hand during fluoroscopy in MIS-TLIF [23]. Funao et al. also reported that the average exposure time for single-level MIS-TLIF was 38.7 s, and the average level of exposure encountered by the surgeon’s hand was 0.32 mSv. They also reported that the exposure to the fingers was about 10 times higher than that to other parts of the body [24]. Fujibayashi et al. also reported damage to fingernails and skin due to radiation exposure [25]. Under these circumstances, evacuation from radiation-exposed areas is suggested to have a beneficial effect in terms of reducing radiation exposure to the hands and fingers [26].

Based on the above findings, attempts were made to develop a minimally invasive and accurate pedicle screw insertion technique using existing spinal surgical instrumentation without direct radiation exposure. First, these issues were dealt with by avoiding direct radiation exposure, not using K-wire guides when inserting percutaneous pedicle screws, and confirming anatomical indicators directly. Furthermore, a tube retractor was used to ensure the same invasiveness as the PPS placement. Second, a self-drilling pin was adapted to capture the cancellous bone inside the pedicle precisely within the narrow tubular retractor. The screw motion of the pin takes advantage of its ability to advance through the softer cancellous bone along with the hard cortical bone. The purpose of this study was to evaluate radiation exposure and accuracy of pedicle screw placement in one-level, minimally invasive transforaminal lumbar interbody fusions (MIS-TLIF) performed using this new procedure.

## 2. Materials and Methods

### 2.1. Study Design

This is a retrospective study of patients who (1) had single-level lumbar disease, (2) underwent single-level MIS-TLIF with pedicle screw placement between July 2018 and February 2020 at Yuki hospital, and (3) had the same fluoroscopy settings. Data including age, gender, weight, body mass index (BMI), primary diagnosis, levels fused, cumulative fluoroscopic time, cumulative area dose product (DAP) and cumulative air kerma (*AK*) (obtained from the DAP monitor system originally installed on the fluoroscope), operating time, extra time required for this procedure, and complications related to screw placement were extracted from each patient’s medical record. To facilitate comparisons with other studies, the entrance skin dose (ESD) for each patient was calculated from the DAP and AK values using the following formula [27,28,29].

$$\mathrm{ESD}=\left(\frac{DAP}{A\left(FID\right)}\right)\cdot {\left(\frac{FID}{FSD}\right)}^{2}\cdot BSF\left(A\left(FID\right)\right){\left(\frac{\mu_{en}}{\rho }\right)}_{Air}^{Tissue}$$

*FID* is the area of the radiation field on the patient skin (cm^2^), *DAP* is the dose measured by a DAP-meter on fluoroscopy (mGy × cm^2^), *FSD* is the distance from X-ray focus to skin (m), FFD is the distance from X-ray focus to film (m), *BSF* is the backscatter factor for the given A (*FID*), and (μen/ρ)Tiss/(μen/ρ)Air is the tissue-to-air mass–energy absorption coefficient ratio. *DAP* is expressed as the product of AK and A (*FID*) [30]. *DAP* is commonly displayed on the fluoroscope screen. AK is added in consideration of the X-ray conditions that change during fluoroscopy and the visual field and is displayed as the irradiation dose at the reference point during the fluoroscopy. Since AK is more accurate than *DAP*, we used AK to calculate ESD.

The effect of patient characteristics on surgical and radiological data was investigated statistically. These results were grouped into categorical data based on patient characteristics and summarized as medians with minimum and maximum values. All univariate analyses were performed using the Mann–Whitney U for the effects of patient age, sex, and BMI (two categories) and the Kruskal–Wallis test for the effects of primary diagnosis and lumbar spine level (three or four categories). Patient age and BMI were changed to nominal variables. Patient age was divided into ≥65 and <65 years old, and BMI was divided into her ≥25 and her <25 kg/m^2^. Statistical analysis was performed using Statcel 4 (OMS Publishing Inc., Saitama, Japan). Statistical significance was defined as *p* < 0.05.

Postoperative CT scans were reviewed for accuracy of placement and categorized using the Gertzbein–Robbins classification [31]. Screws entirely within the pedicle are Grade A; a breach of less than 2 mm is Grade B; a breach of 2 mm or more and less than 4 mm is Grade C; a breach of 4 mm or more and less than 6 mm is Grade D; and a breach greater than 6 mm is Grade E. Simple descriptive statistics were used to analyze and describe the data. This study was approved by the Institutional Review Board.

### 2.2. Operating Room Workflow

**Figure 1:**
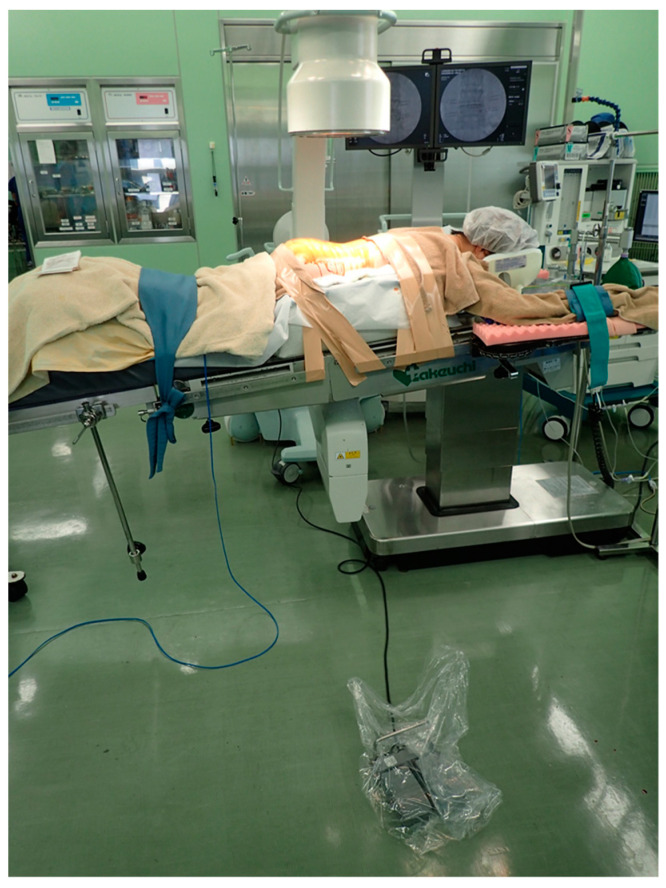
Operating room setup (the foot switch is at least 1 m away from the operating table).

For each operation, the patient was positioned prone on a radiolucent spinal operating table after induction of general anesthesia and endotracheal intubation. Disposable injection needles were placed at the outer edge of the pedicles under fluoroscopy after preoperative skin preparation. Surgeons wore a lead apron and thyroid shield, placed the foot switch at a distance of at least 1 m, and confirmed placement by one-shot anteroposterior (AP) fluoroscopy (Figure 1).

**Figure 2:**
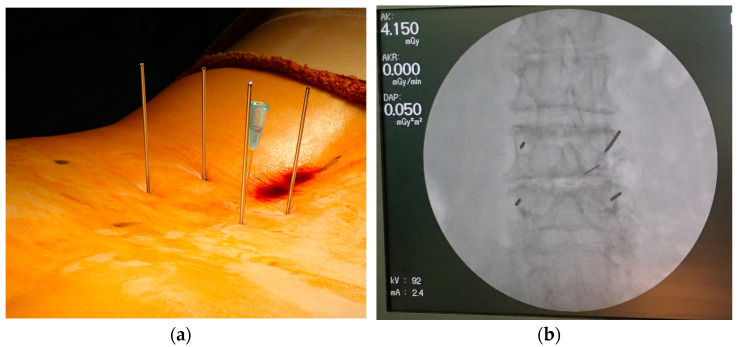
(**a**) Insertion of K-wires at outer edge of pedicles; (**b**) confirmation of K-wire placement under fluoroscopy.

A disposable needle was adjusted semi-blindly to avoid direct radiation exposure. With reference to each disposable needle, 1.5 mm K-wires were firmly placed at the outer edge of pedicles (Figure 2a). Disposable needles were adjusted hemianopically to avoid direct radiation exposure. Referring to each disposable needle, a 1.5 mm K-wire was placed firmly on the outer edge of the pedicle (Figure 2a). Even at this time, the K-wire installation was performed semi-blindly. Fine adjustment of the K-wire insertion site was performed while confirming the one-shot image after evacuating the hands and fingers from the radiation exposure. The orientation of the K-wires was confirmed by AP and lateral fluoroscopy (Figure 2b). The OPESCOPE ACTENO C-arm system (Shimadzu, Kyoto, Japan) was used for all cases. The settings on the fluoroscopy unit were changed from standard settings to low dose and pulse (7.5 p/s) mode. Since it was necessary to keep the imaging conditions constant to estimate ESD, irradiation was performed from under the operating table in principle.

### 2.3. Surgical Technique

**Figure 3:**
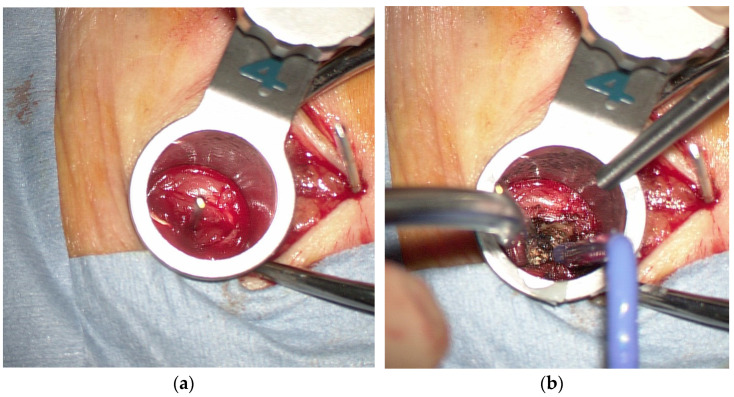
(**a**) A tubular retractor placed over the outside of the facet joint along the K-wire; (**b**) exposure of the lateral margin of the facet joint.

**Figure 4:**
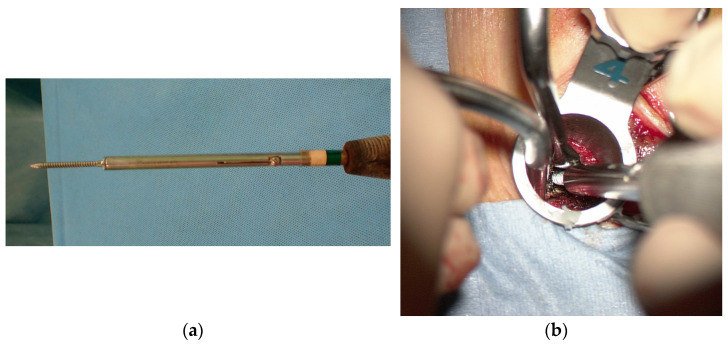
(**a**) A self-drilling pin; (**b**) a self-drilling pin inserted into the pedicle.

After preparation and surgical draping, a skin incision approximately 3 cm long was made between the upper and lower K-wires on the symptomatic side for laminectomy. On the other side of the laminectomy, two 1.5 cm skin incisions were made at each K-wire insertion site for screw insertion. To prepare for insertion of the pedicle screw prior to laminectomy, serial dilators and the final tubular retractor (16 mm or 18 mm diameter) were placed over the outside of the facet joint along the K-wire (Figure 3a). After removing the K-wire in the tubular retractor under a microscope (Figure 3b), a self-drilling pin (Figure 4a) was inserted into the pedicle from the lateral edge of the facet joint instead of the K-wire guide (Figure 4b).

**Figure 5:**
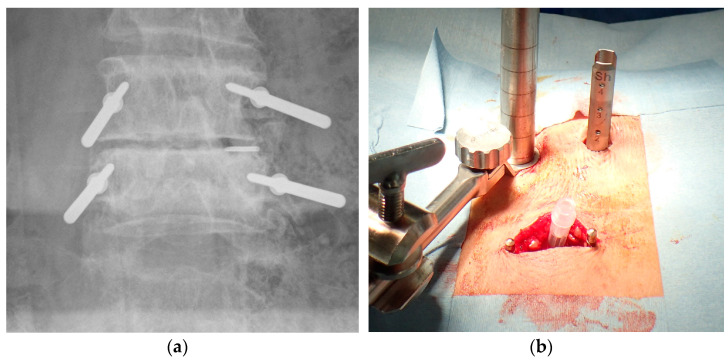
(**a**) Posteroanterior radiograph showing direction of self-drilling pins; (**b**) Pedicle screw insertion into the opposite side of the laminectomy via the tubular retractor.

Four self-drilling pins were inserted into the pedicle in the same manner. After confirming the position of the self-drilling pins on a two-way radiograph (Figure 5a), pedicle screws were inserted on the side opposite to the laminectomy using the tubular retractor under a microscope for restoration of disc height (Figure 5b). The length of the pedicle screw was determined using a depth gauge after tapping the inside of the pedicle under the microscope. Self-drilling pins on the laminectomy side were replaced with small pedicle markers so as not to interfere with laminectomy. If self-drilling pin insertion was difficult during this process, the surgeon and staff left the room, and the direction of the self-drilling pin was confirmed and corrected by one-shot AP fluoroscopy.

**Figure 6:**
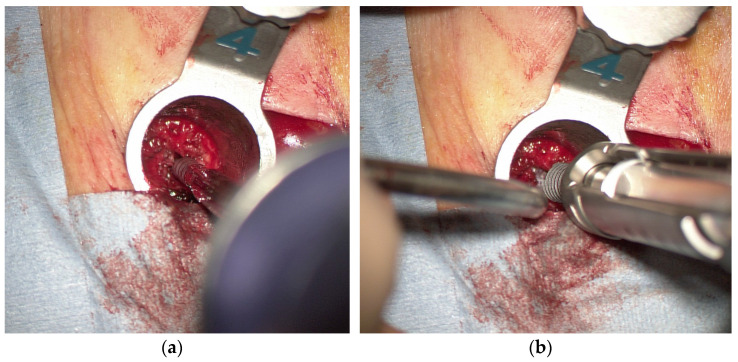
(**a**,**b**) Insertion of the pedicle screw under the microscope.

Under the microscope, a laminectomy and complete facetectomy were performed on the symptomatic side using a tubular retractor (22 mm diameter), nerve roots were decompressed, and the disc space was accessed. After restoration of the disc height using distractors and completion of the discectomy, an interbody spacer was inserted into the disc space with autologous bone, artificial bone, and 10 mL of bone marrow fluid. After placement of the interbody, pedicle screws on the laminectomy side (Figure 6a,b) were inserted in the same manner as described, and the rod was fastened. All patients underwent postoperative AP and lateral radiography to evaluate instrumentation after surgery.

## 3. Results

### 3.1. Patient Characteristics

Twenty-four patients underwent single-level MIS TLIF, resulting in the placement of 96 pedicle screws. There were 15 women and 9 men, with an average age of 64.8 years (range 24–88 years). The mean body mass index was 25.5 kg/m^2^ (range 17.3–32.9 kg/m^2^). Indications for surgery included spondylolisthesis (13 patients), degenerative disc disease with radiculopathy (5 patients), and disc herniation (6 patients). Operative levels included 1 at L1-2, 5 at L3-4, 14 at L4-5, and 4 at L5-S1 (Table 1).

### 3.2. Radiation Time and Radiation Dose during Surgery

The mean operating time was 201.8 min (range: 145–246 min). The mean fluoroscopic time was 26.8 s (range: 8–56 s). The mean radiation dose of DAP was 0.0706 mGy∗m^2^ (range: 0.018–0.133 mGy∗m^2^). The mean radiation dose of AK was 6.0 mGy (range: 1.071–21.74 mGy). The mean radiation dose of ESD was 11.31 mGy (range: 2.199–44.64 mGy; Table 2). Statistically, there was no effect of various patient characteristics on each result (Table 3).

### 3.3. Pedicle Screw Insertion Accuracy

Regarding insertion accuracy, A corresponds to 93/96 (96.88%), B to 0/96 (0%), C to 2/112 (2.08%), and D to 1/96 (1.04%; Table 4). 

### 3.4. Adverse Events

**Figure 7:**
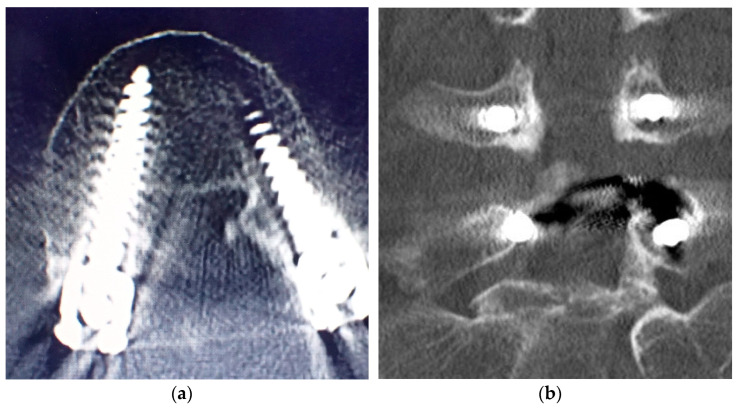
(**a**,**b**) Computed tomography scan of the screw deviation to the medial pedicle wall in grade D breach.

In one patient with a grade D breach (Figure 7a,b), screw deviation of the medial pedicle wall with numbness of the right foot developed postoperatively. After reoperation, the symptoms resolved, and no residual neuropathy was observed in this patient. The extra time required for this new procedure was also considered. The extra time was defined from the start of surgery to the completion of intraoperative radiography of the pedicle marker. The average time was 39 min (range: 16–69 min; Table 2) per patient, and patient characteristics had no effect on this result (Table 3).

## 4. Discussion

Even compared to previous reports, the results of this study suggest that pedicle screw placement using a tube retractor, microscope, and a self-drilling pin allowed for reduced fluoroscopic radiation exposure and ensured sufficient accuracy of screw placement. In addition, there are three benefits to this new technique. The first is that this procedure does not cause direct radiation exposure. It is possible to minimize exposure to hands and fingers. Second, the tube retractor enables reduction in the invasiveness of pedicle screw insertion to the same extent as PPS placement. Third, pedicle search using screw pins contributes to improved pedicle screw insertion accuracy.

Although there are reports of reducing radiation exposure during PPS placement under fluoroscopy [16,17,18,19,20], direct radiation exposure to hands and fingers during surgery remains a major problem [21,22,23,24,25]. In this new procedure, the radiation exposure range is approached only during the insertion of preoperative K-wire into the outer edge of the pedicle. In this situation, the amount of radiation exposure is very small because the surgeon wears a lead apron and a thyroid shield, removes hands and fingers from the radiation field, and steps on the exposure footswitch at a distance of at least 1 m. Radiation measurements were attempted using a radiation detector outside the lead apron. Radiation was not detected (data not shown). The procedure described herein may be beneficial with a greater reduction in radiation exposure, limiting direct radiation exposure faced by surgeons and medical staff.

The skin incision for placing the tube retractor in this procedure is about 15 mm long. The inner diameter of the 16-18 mm diameter tube retractor is approximately the same as the outer diameter of the pedicle screwdriver (Figure 6b). Pedicle screw insertion is possible without the need for greater invasiveness, and it is as invasive as PPS placement. This is enough space for the screw pin usage described below. Use with a microscope together contributes to supporting a deep field of view even without using fluoroscopy.

The result for Grade A placement of percutaneous pedicle screw insertion was 97%. This result is comparable to other studies [32,33,34,35] and can be attributed to the use of self-drilling pins instead of K-wire guides. The self-drilling is pin slowly rotated in the cancellous bone to search for the direction of the pedicle while creating a pilot hole. This serves to accurately capture the pedicle in a narrow tubular retractor without direct radiation exposure. Even if it deviates, given that the self-drilling pin moves at a low speed, the risk of nerve root damage is low. This procedure is feasible and safe in terms of screw insertion accuracy.

**Figure 8:**
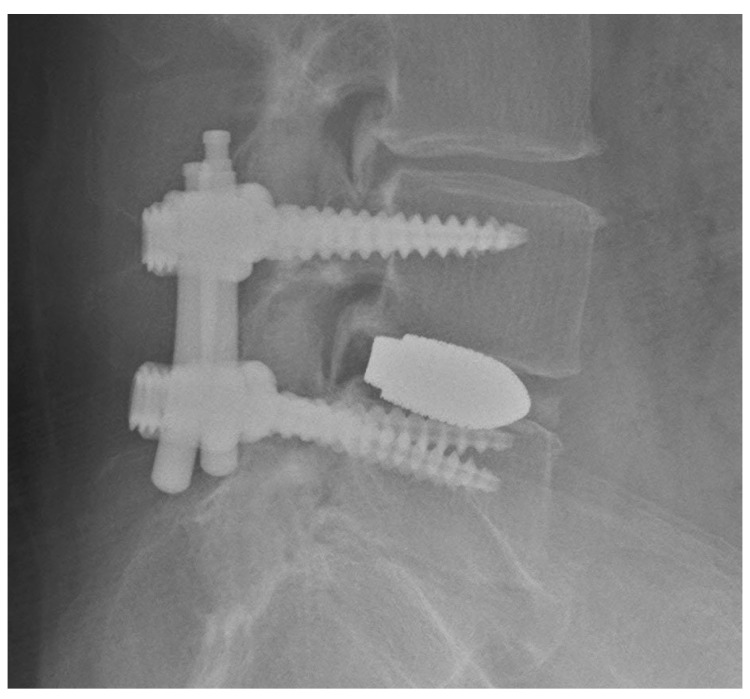
Lateral radiograph of ossification of the posterior longitudinal ligament of the lumbar spine.

There are two issues that still need to be considered. The first is the medial deviation of the pedicle of the screw in one of the cases with a Grade D result. This case was a patient with strong osteophyte changes in the facet joints with ossification of the posterior longitudinal ligament of the lumbar spine (Figure 8).

Proper insertion may be difficult in cases with strong osteophyte changes in the facet joints and cases with osteosclerosis of the pedicle, even with the Jamshidi needle procedure. In our case, it was thought that the self-drilling pin was deflected inward by insertion from above the osteophyte. Based on this experience, we are currently adjusting the insertion point by checking the lateral edge of the facet joint to avoid the osteophyte. If the self-drilling pin is hard to insert, the image is confirmed by changing the insertion direction. In subsequent cases, no medial deviations have yet been observed. As an improvement, the combined use of electromyography may be effective to prevent complications [36,37]. The second issue is the extension of operating time due to the addition of this procedure. The time taken was significantly extended to 39 min on average. This was because the technique had not yet been mastered at the beginning. Furthermore, other major causes were that the process was complicated, and the equipment was not sophisticated. In the future, in order to develop the procedure and conduct multicenter research, it is considered necessary to simplify the procedure and refine the equipment. Apart from these, there were no other significant complications such as large vessel damage, intra-abdominal organs, increased bleeding, or infections.

Li et al. performed pedicle screw placement using a tubular retractor [38]. However, their procedure describing insertion of the Jamshidi needles using a tubular retractor is different from that described here. In that study, the reduction in radiation exposure and accuracy of screw insertion were not evaluated, preventing comparison with the technique described herein. Further details of their technique are awaited in the future.

This study demonstrates that radiation exposure during minimally invasive pedicle screw placement can be reduced, ensuring sufficient screw insertion accuracy, even with a procedure that does not use direct fluoroscopy to protect the hands and fingers. The present study also demonstrates the feasibility of using existing spinal surgical instrumentation to achieve these results. The present study has several limitations, including being performed at a single institution, with a small number of patients, and being a single-arm study. It will be necessary to simplify the procedure and conduct research at multiple facilities. A future goal is to use multiple devices that use EMG and ultrasound to ensure accuracy and safety in less time [39,40].

## 5. Conclusions

A tubular retractor under a microscope and self-drilling pins provide high accuracy for minimally invasive pedicle screw placement, decreasing the radiation exposure caused by fluoroscopy faced by surgeons, medical staff, and patients. In particular, it prevents direct radiation exposure to hands and fingers. Furthermore, it is economical and does not require the purchase of new equipment. It is a safe and feasible procedure, although further study is needed to confirm these preliminary results.

## Figures and Tables

**Table 1:** Patient demographic and treatment information.

Characteristic	Value (%)
Number of patients	24
Mean patient age in years (range)	64.8 (24–88)
Men (%)	9 (37.5)
Women (%)	15 (62.5)
Body Mass Index (kg/m^2^) (range)	25.5 (17.3–32.9)
Primary diagnosis	
Spondylolisthesis	13 (51)
Degenerative disc disease	5 (22)
Disc herniation	6 (25)
Lumbar level	
L1/2	1
L3/4	5
L4/5	14
L5/S1	4

**Table 2:** Surgery and radiation data for 24 patients who underwent MIS TLIF.

Factor	Mean Value (Range)
Operating time (minutes)	201.8 (145–246)
Fluoroscopic time/case (seconds)	26.8 (8–56)
Radiation dose of DAP (mGy∗m^2^)	0.0706 (0.018–0.133)
Radiation dose of AK (mGy)	6.0 (1.071–21.74)
Radiation dose of ESD (mGy)	11.31 (2.199–44.64)
Extra time required for this procedure (minutes)	39 (16–69)

MIS TLIF: minimally invasive transforaminal lumbar interbody fusions. DAP: area dose product. AK: air kerma. ESD: entrance skin dose.

**Table 3:** Statistical examination of surgery and radiation data according to patient characteristics.

Age	<65 Years Old (*n* = 12)		≥65 Years Old (*n* = 12)		
Fluoroscopic time (seconds)	26.5 (8–56)		24.5 (15–49)		*p* = 0.582
Radiation dose of DAP (mGy∗m^2^)	0.061 (0.018–0.128)		0.058 (0.034–0.26)		*p* = 0.931
Radiation dose of ESD (mGy)	9.884 (3.002–21.928)		9.823 (2.199–44.637)		*p* = 0.908
Extra time required for this procedure (minutes)	40 (27–71)		33 (16–64)		*p* = 0.111
**Gender**	**Men (*n* = 9)**		**Women (*n* = 15)**		
Fluoroscopic time (seconds)	24 (8–49)		27 (15–56)		*p* = 0.881
Radiation dose of DAP (mGy∗m^2^)	0.057 (0.018–0.102)		0.061 (0.032–0.26)		*p* = 0.811
Radiation dose of ESD (mGy)	9.747 (3.002–19.341)		0.447 (2.199–44.637)		*p* = 0.743
Extra time required for this procedure (minutes)	33 (27–48)		39 (16–71)		*p* = 0.367
**BMI**	**<25 kg/m^2^ (*n* = 12)**		**≥25 kg/m^2^ (*n* = 12)**		
Fluoroscopic time (seconds)	24 (15–49)		26 (8–56)		*p* = 0.862
Radiation dose of DAP (mGy∗m^2^)	0.0495 (0.032–0.102)		0.066 (0.018–0.26)		*p* = 0.119
Radiation dose of ESD (mGy)	9.232 (5.433–19.341)		10.59 (2.199–44.637)		*p* = 0.299
Extra time required for this procedure (minutes)	37 (16–69)		33 (18–71)		*p* = 0.977
**Primary diagnosis**	**Spondylolisthesis** **(*n* = 13)**		**Degenerative disc disease (*n* = 5)**	**Disc herniation (*n* = 6)**	
Fluoroscopic time (seconds)	25 (15–56)		28 (15–38)	24.5 (8–49)	*p* = 0.798
Radiation dose of DAP (mGy∗m^2^)	0.058 (0.032–0.26)		0.063 (0.034–0.088)	0.0535 (0.018–0.102)	*p* = 0.907
Radiation dose of ESD (mGy)	9.878 (2.199–44.637)		10.734 (5.819–14.999)	8.951 (3.002–19.341)	*p* = 0.655
Extra time required for this procedure (minutes)	33 (16–71)		39 (30–64)	31.5 (27–47)	*p* = 0.444
**Lumbar Level**	**L1/2 (*n* = 1)**	**L3/4 (*n* = 5)**	**L4/5 (*n* = 14)**	**L5S1 (*n* = 4)**	
Fluoroscopic time (seconds)	24	27 (15–49)	26 (15–56)	24.5 (8–28)	*p* = 0.896
Radiation dose of DAP (mGy∗m^2^)	0.044	0.061 (0.034–0.102)	0.0635 (0.032–0.26)	0.058 (0.018–0.063)	*p* = 0.662
Radiation dose of ESD (mGy)	7.593	10.447 (5.819–19.341)	9.812 (2.199–44.637)	9.894 (3.002–10.734)	*p* = 0.834
Extra time required for this procedure (minutes)	47	30 (9–64)	34.5 (16–71)	39.5 (30–63)	*p* = 0.714

Data are presented as the median with the minimum and maximum values. BMI: body mass index.

**Table 4:** Pedicle screw breach rate (total and per vertebral level).

Vertebral Level	Screws Per Vertebral Level	Number of Breaches	Direction of Breach (*n*)	Grade of Breach (*n*)	Breach Rate (%)
L-1	2	0			0
L-2	2	0			0
L-3	10	0			0
L-4	38	3	Medial (1), Lateral (2)	C (2), D (1)	3.2
L-5	36	0			
S-1	8	0			0
Total	96	3			3.2

## Data Availability

The dataset generated during the current study is available from the corresponding authors on reasonable request.
